# Prevalence of stress, anxiety, depression among the general population during the COVID-19 pandemic: a systematic review and meta-analysis

**DOI:** 10.1186/s12992-020-00589-w

**Published:** 2020-07-06

**Authors:** Nader Salari, Amin Hosseinian-Far, Rostam Jalali, Aliakbar Vaisi-Raygani, Shna Rasoulpoor, Masoud Mohammadi, Shabnam Rasoulpoor, Behnam Khaledi-Paveh

**Affiliations:** 1 Department of Biostatistics, School of Health, Kermanshah University of Medical Sciences, Kermanshah, Iran; 2 Sleep Disorders Research Center, Kermanshah University of Medical Sciences, Kermanshah, Iran; 3 Department of Business Systems & Operations, University of Northampton, Northampton, UK; 4 Department of Nursing, School of Nursing and Midwifery, Kermanshah University of Medical Sciences, Kermanshah, Iran; 5 Department of Biology, Islamic Azad University Urmia, Urmia, Iran

**Keywords:** COVID-19, Coronavirus, Prevalence, Stress, Anxiety, Depression, General population, Meta-analysis, Systematic review

## Abstract

**Background:**

The COVID-19 pandemic has had a significant impact on public mental health. Therefore, monitoring and oversight of the population mental health during crises such as a panedmic is an immediate priority. The aim of this study is to analyze the existing research works and findings in relation to the prevalence of stress, anxiety and depression in the general population during the COVID-19 pandemic.

**Method:**

In this systematic review and meta-analysis, articles that have focused on stress and anxiety prevalence among the general population during the COVID-19 pandemic were searched in the Science Direct, Embase, Scopus, PubMed, Web of Science (ISI) and Google Scholar databases, without a lower time limit and until May 2020. In order to perform a meta-analysis of the collected studies, the random effects model was used, and the heterogeneity of studies was investigated using the I^2^ index. Moreover. data analysis was conducted using the Comprehensive Meta-Analysis (CMA) software.

**Results:**

The prevalence of stress in 5 studies with a total sample size of 9074 is obtained as 29.6% (95% confidence limit: 24.3–35.4), the prevalence of anxiety in 17 studies with a sample size of 63,439 as 31.9% (95% confidence interval: 27.5–36.7), and the prevalence of depression in 14 studies with a sample size of 44,531 people as 33.7% (95% confidence interval: 27.5–40.6).

**Conclusion:**

COVID-19 not only causes physical health concerns but also results in a number of psychological disorders. The spread of the new coronavirus can impact the mental health of people in different communities. Thus, it is essential to preserve the mental health of individuals and to develop psychological interventions that can improve the mental health of vulnerable groups during the COVID-19 pandemic.

## Background

In December 2019, in the city of Wuhan, China, unusual cases of patients with pneumonia caused by the new Coronavirus (COVID-19) were reported [1], and the spread of the virus swiftly became a global health threat [2]. There have been several viral diseases in the past 20 years including Severe Acute Respiratory Syndrome (SARS) in 2003, influenza virus with the H1N1 subtype in 2009, Middle East Respiratory Syndrome (MERS) in 2012, and Ebola virus in 2014 [3–5].

Although COVID-19 is a new strain of coronaviruses, it is known to cause diseases ranging from cold to more severe illnesses such as SARS and MERS [5]. Symptoms of the Coronavirus infection include fever, chills, cough, sore throat, myalgia, nausea and vomiting, and diarrhea. Men with a history of underlying diseases are more likely to be infected with the virus and would experience worse outcomes [6]. Severe cases of the disease can lead to heart, and respiratory failure, acute respiratory syndrome, or even death [7]. In addition to the physical impacts, COVID-19 can have serious effects on people’s mental health [8]. A wide range of psychological outcomes have been observed during the Virus outbreak, at individual, community, national, and international levels. At the individual level, people are more likely to experience fear of getting sick or dying, feeling helpless, and being stereotyped by others [9]. The pandemic has had a harmful effect on the public mental health which can even lead to psychological crises [10]. Early identification of individuals in the early stages of a psychological disorder makes the intervention strategies more effective. Health crises such the COVID-19 pandemic lead to psychological changes, not only in the medical workers, but also in the citizens, and such psychological changes are instigated by fear, anxiety, depression, or insecurity [11].

Nervousness and anxiety in a society affect everyone to a large extent. Recent evidence suggests that people who are kept in isolation and quarantine experience significant levels of anxiety, anger, confusion, and stress [12]. At large, all of the studies that have examined the psychological disorders during the COVID-19 pandemic have reported that the affected individuals show several symptoms of mental trauma, such as emotional distress, depression, stress, mood swings, irritability, insomnia, attention deficit hyperactivity disorder, post-traumatic stress, and anger [12–14]. Research has also shown that frequent media exposure may cause distress [15]. Nevertheless, in the current situation, it is challenging to accurately predict the psychological and emotional consequences of COVID-19. Studies conducted in China, the first country that was affected by this recent Virus spread, show that people’s fear of the unknown nature of the Virus can lead to mental disorders [16].

Due to the pathogenicity of the virus, the rate of spread, the resulting high mortality rate, COVID-19 may affect the mental health of individuals at several layers of society, ranging from the infected patients, and health care workers, to families, children, students, patients with mental illness, and even workers in other sectors [17–19].

**Fig. 1:**
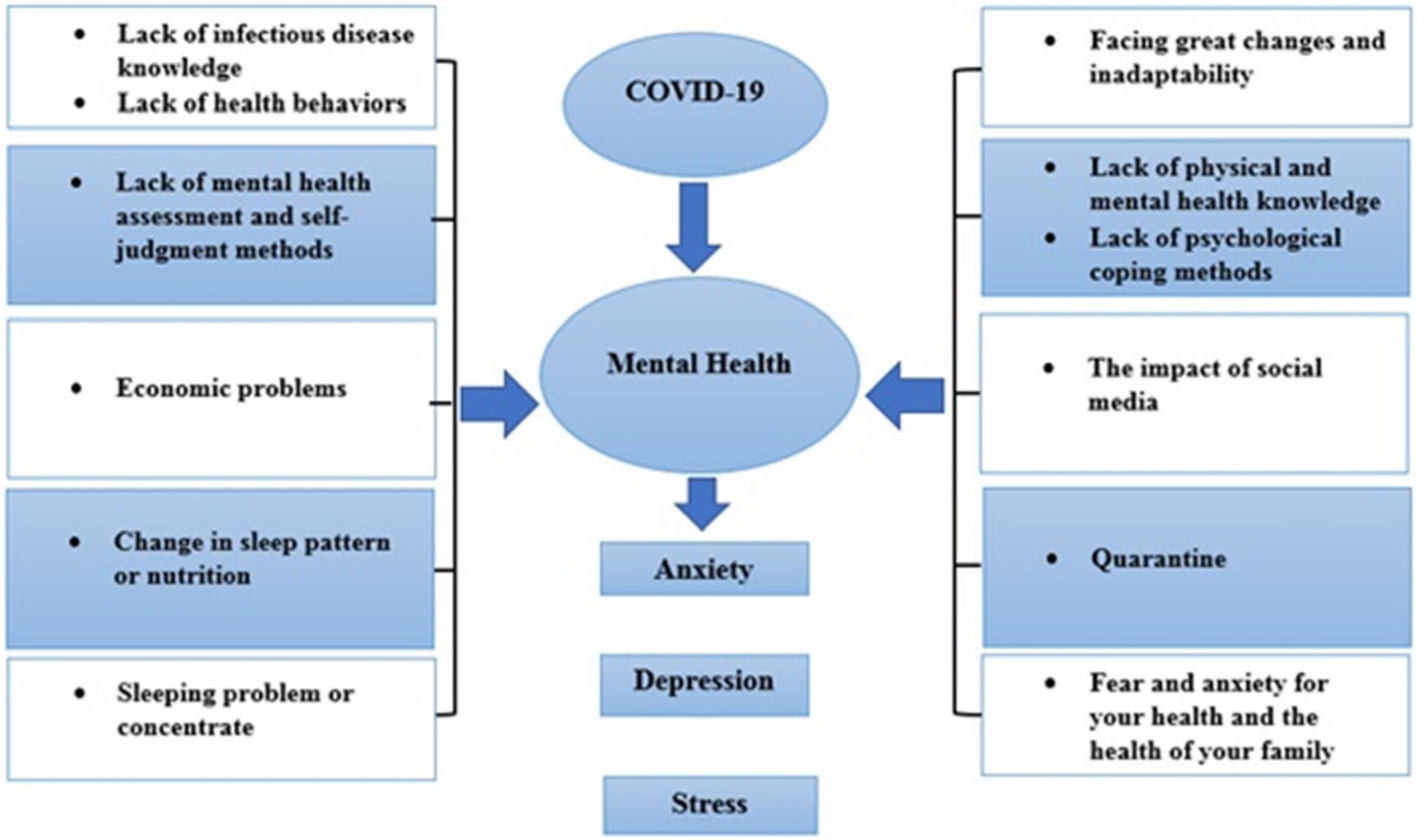
Impacts of the COVID-19 pandemic on mental health

Considering several reported psychological consequences of COVID-19 and its spread (Fig. 1), and the lack of general statistics on the topic globally, we decided to conduct a systematic review of the existing studies in this field, with a view to providing a holistic, yet comprehensive statistics on the impact of the Virus on general population mental health. The aim of this study is to examine and systematically review and analyze the literature and their reported results related to the impacts of COVID-19 on the prevalence of stress, anxiety, and depression.

## Method

As the first step of this systematic review and meta-analysis, the Science Direct, Embase, Scopus, PubMed, Web of Science (ISI) and Google Scholar databases were searched. To identify the articles, the search terms of Coronavirus, COVID-19, 2019-ncov, SARS-cov-2, Mental illness, Mental health problem, Distress, Anxiety, Depression, and all the possible combinations of these keywords were used.

(((((((((((((Coronavirus [Title/Abstract]) OR (COVID-19[Title/Abstract])) OR (2019-ncov [Title/Abstract])) AND (SARS-cov-2[Title/Abstract])) AND (Mental illness [Title/Abstract])) OR (Mental health problem [Title/Abstract])) AND (Anxiety [Title/Abstract])) AND (Social Anxiety [Title/Abstract])) OR (Anxiety Disorders [Title/Abstract])) AND (Depression [Title/Abstract])) OR (Emotional Depression [Title/Abstract])) OR (Depressive Symptoms [Title/Abstract]))))))))))))

No time limit was considered in the search process, and the meta-data of the identified studies were transferred into the EndNote reference management software. In order to maximize the comprehensiveness of the search, the lists of references used within all the collected articles were manually reviewed.

### Inclusion and exclusion criteria

The criteria for entering the systematic review included: 1- Studies that examined the prevalence of stress, anxiety, depression among the general population during the COVID-19 pandemic. 2- Studies that were observational (i.e. non-interventional studies) 3- Studies that their full text was available. The criteria for excluding a study were: 1- Unrelated research works, 2- Studies without sufficient data, 3- Duplicate sources, 4-Pieces of research with unclear methods 5- Interventional studies 6- Case reports, and 7- Articles that their full text was not available.

### Study selection

Initially, duplicate articles that were repeatedly found in various databases were removed. Then, a title list of all the remaining articles was prepared, so that the articles could be filtered out during the evaluation phase in a structured way. As part of the first stage of the systematic review process, i.e. screening, the title and abstract of the remaining articles were carefully examined, and a number of articles were removed considering the inclusion and exclusion criteria. In the second stage, i.e. eligibility evaluation, the full text of the studies, remaining from the screening stage, were thoroughly examined according to the criteria, and similarly, a number of other unrelated studies were excluded. To prevent subjectivity, article review and data extraction activities were performed by two reviewers, independently. If an article was not included, the reason for excluding it was mentioned. In cases where there was a disagreement between the two reviewers, a third person reviewed the article. Seventeen studies entered the third stage, i.e. quality evaluation.

### Quality evaluation

In order to examine the quality of the remaining articles (i.e. methodological validity and results), a checklist appropriate to the type of study was adopted. STROBE checklists are commonly used to critique and evaluate the quality of observational studies. The checklist consists of six scales/general sections that are: title, abstract, introduction, methods, results, and discussion. Some of these scales have subscales, resulting in a total of 32 fields (subscales). In fact, these 32 fields represent different methodological aspects of a piece of research. Examples of subscales include title, problem statement, study objectives, study type, statistical population, sampling method, sample size, the definition of variables and procedures, data collection method(s), statistical analysis techniques, and findings. Accordingly, the maximum score that can be obtained during the quality evaluation phase and using the STROBE checklist is 32. By considering the score of 16 as the cut-off point, any article with a score of 16 or above is considered as a medium or a high-quality article [20]. Sixteen papers obtained a score below 16, denoting a low methodological quality, and were therefore excluded from the study. In the present study, following the quality evaluation by means of the STROBE checklist, 17 papers, with a medium or high quality, entered the systematic review and meta-analysis phases.

### Data extraction

Data of from all the final studies were extracted using a different pre-prepared checklist. The items on the checklist included: article title, first author’s name, year of publication, place of study, sample size, assessment method, gender, type of study, the prevalence of depression, anxiety, and stress.

### Statistical analysis

The I^2^ (%) test was used to assess the heterogeneity of the selected research works. In order to assess publication bias, due to the high volume of samples that entered the study, the Egger’s test was conducted with the significance level of 0.05, and the corresponding Forest plots were drawn. Data analysis was performed using the Comprehensive Meta-Analysis (CMA version 2.0) software.

## Results

**Fig. 2:**
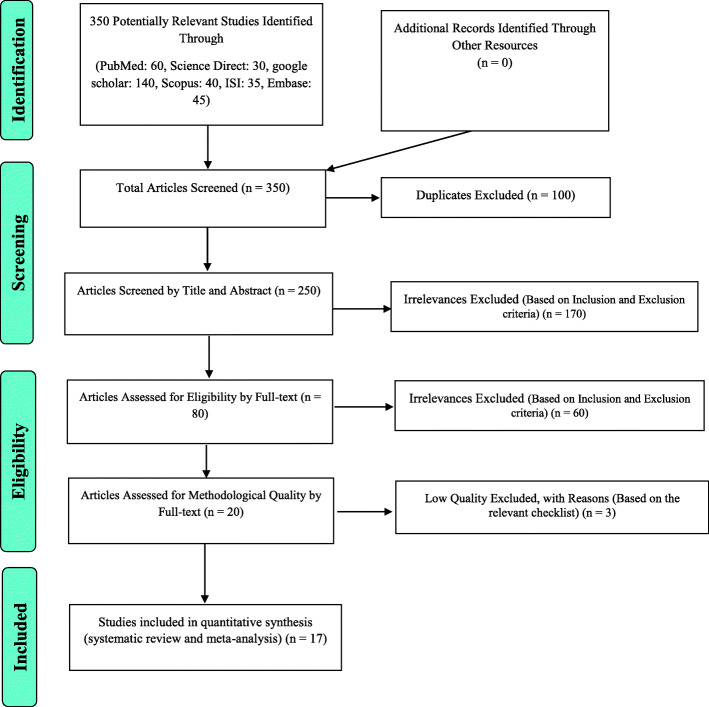
PRISMA (2009) flow diagram demonstrating the stages for sieving articles in this systematic review and meta-analysis

In this work, the prevalence of stress and anxiety among general population during the COVID-19 pandemic was assessed. Articles with this focus were collected with no lower time limit and until May 2020 and were systematically reviewed according to the PRISMA guidelines. Following the initial search, 350 possible related articles were identified and transferred to the reference management software, EndNote. Of the 350 studies identified, 100 were duplicates, and therefore excluded. At the screening stage, out of the remaining 250 studies, 170 articles were removed after assessing their title and abstract and considering the inclusion and exclusion criteria. At the eligibility evaluation phase, out of the remaining 80 studies, 60 articles were removed after the examination of their full text, and similarly by considering the inclusion and exclusion criteria. At the quality evaluation stage, through the evaluation of the full text of the articles, and based on the score obtained from the STROBE checklist for each paper, out of the remaining 20 studies, 3 studies, that were assessed as low methodological quality works, were eliminated, and finally 17 cross-sectional studies reached the final analysis stage (please see Fig. 2). Details and characteristics of these articles are also provided in Table 1.

**Table 1:** Summary of characteristics of the included studies

Author [Reference]	Year	Region	Study population	Male%	Assessment	STROBE score	sampling method	Cut off	Outcomes (sample size)		
									Depression % (n)	Anxiety % (n)	Stress % (n)
A Moghanibashi-Mansourieh [21]	2020	Iran	10,754	34.2%	DASS-21	28	online survey	A > 7	N.A.	50.9% (5472)	N.A.
MZ Ahmed.et al. [22]	2020	China	1074	53.2%	BAI BDI-II	23	online survey	≥8 ≥ 14	37.1% (399)	29% (312)	N.A.
C Wang.et al. [23]	2020	China	1210	32.7%	DASS-21	22	online survey	A > 7 D > 9 S > 10	30.3% (367)	36.4% (440)	32.1% (389)
W Cao.et al. [24]	2020	China	7143	30.35%	GAD-7	20	cluster sampling	≥5	N.A.	24.9% (1776)	N.A.
Y Huang. et al. [25]	2020	China	7236	45.4%	GAD-7 CES-D	18	web-based survey	≥9 ≥ 28	20.1% (1454)	35.1% (2540)	N.A.
M Ueda. et al. [26]	2020	Japan	1000	49.6%	GAD-7 PHQ-9	25	online survey	≥10 ≥ 10	43.1% (431)	33.2% (332)	N.A.
D Liu.et al. [27]	2020	China	14,592	31.6%	GAD-7 PHQ-9	26	online survey	N.A.	53.5% (7503)	44.6% (6196)	N.A.
SJ Zhou .et al. [23]	2020	China	8079	46.5%	GAD-7 PHQ-9	26	online survey	> 4 > 4	43.7% (3533)	37.4% (3020)	N.A.
A Sigdel. et al. [28]	2020	Nepal	349	54.2%	GAD-7 PHQ-9	29	online survey	≥10 ≥ 10	34% (119)	31% (109)	N.A.
SSH Kazmi. et al. [29]	2020	India	1000	38%	DASS-21	19	online survey	A > 7 D > 9 S > 10	38.9% (389)	43% (430)	35.7% (357)
N Othman. et al. [30]	2020	Iraq	548	49.6%	DASS-21	19	online survey	A > 7 D > 9 S > 10	44.9% (246)	47.1% (258)	17.5% (96)
Y Wang. et al. [31]	2020	China	600	44.5%	SAS SDS	19	online survey	≥50 ≥ 50	17.17% (103)	6.33% (38)	N.A.
M Qian. et al. [32]	2020	China	1011	50.44%	GAD-7	28	elephone survey via random digital dialing	≥10	N.A.	26.6% (269)	N.A.
M Shevlin. et al. [33]	2020	UK	2025	48%	GAD-7 PHQ-9	22	online survey (quota sampling)	≥10 ≥ 10	22.12% (448)	21.63% (438)	N.A.
P Odriozola-González. et al. [34]	2020	Spain	3550	35.1%	DASS-21	24	social media	A > 6 D > 9 S > 10	44.1% (1566)	32.4% (1150)	37% (1314)
SF Agberotimi. et al. [35]	2020	Nigeria	502	53.6%	GAD-7 PHQ-9	29	Respondent-Driven Sampling (RDS) technique and Random Survey Sampling (RSS)	> 5 ≥ 10	23.5% (118)	49.6% (249)	N.A.
C Mazza. et al. [36]	2020	Italy	2766	28.3%	DASS-21	27	online survey	A > 6 D > 9 S > 10	32.8% (906)	18.7% (517)	27.2% (752)

*DASS-21* The Depression, Anxiety and Stress Scale, *GAD-7* Generalized Anxiety Disorder 7-item, *PHQ-9* Patient Health Questionnaire, *SAS* Zung Self-Rating Anxiety Scale, *SDS* Zung Self-Rating Depression Scale, *BAI* the Beck Anxiety Inventory, *BDI* Beck Depression Inventory, *CES-D* Center for Epidemiologic Studies Depression Scale

### Investigating heterogeneity and publication Bias

**Fig. 3:**
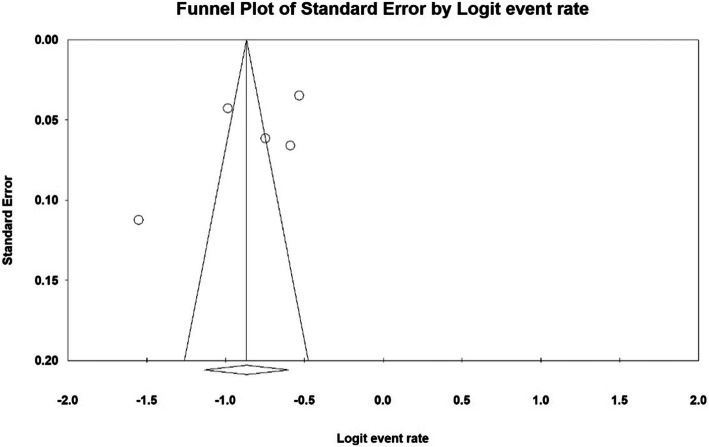
Funnel plot of results of prevalence of stress among the general population during the COVID-19 pandemic

**Fig. 4:**
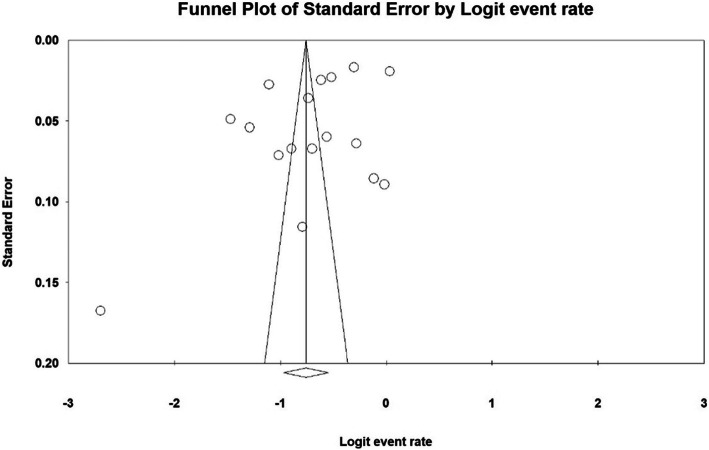
Funnel plot of results of prevalence of anxiety among the general population during the COVID-19 pandemic

**Fig. 5:**
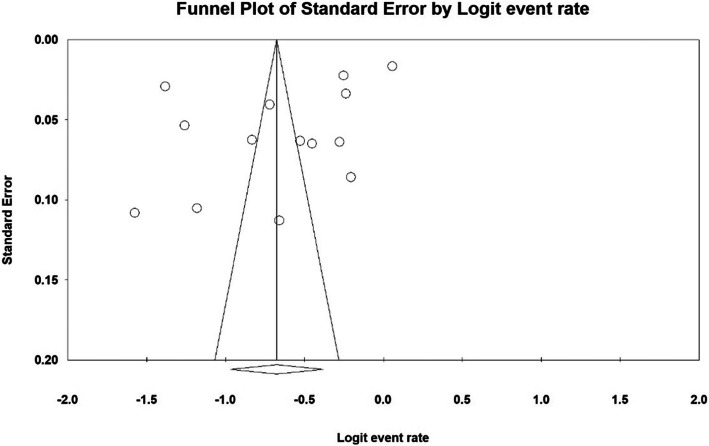
Funnel plot of results of prevalence of depression among the general population during the COVID-19 pandemic

To investigate the heterogeneity of the studies, the I^2^ (%) indices for the prevalence of stress (I^2^: 96.8%), anxiety (I^2^: 99.3%) and depression (I^2^: 99.4%) were obtained. Due to the high heterogeneity in the studies, the random effects model was used in the analysis of findings. To examine publication bias in the collected articles, the Egger’s test indices were obtained for the prevalence of stress (p: 0.304) (Fig. 3), anxiety (p: 0.064) (Fig. 4), and depression (p: 0.073) (Fig. 5), indicating that publication bias was not significant for any of the three clinical symptoms.

### Meta-analysis

**Fig. 6:**
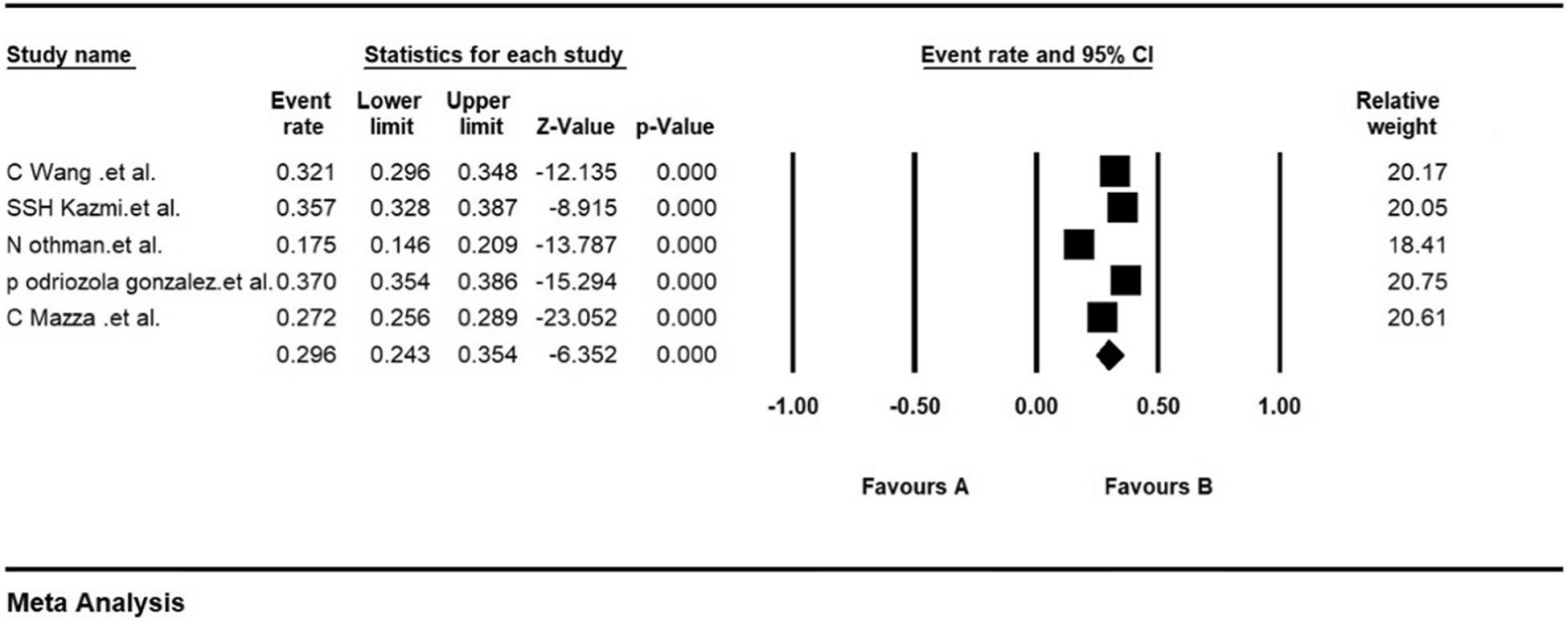
The prevalence of stress in the studies based on the random effects model

**Fig. 7:**
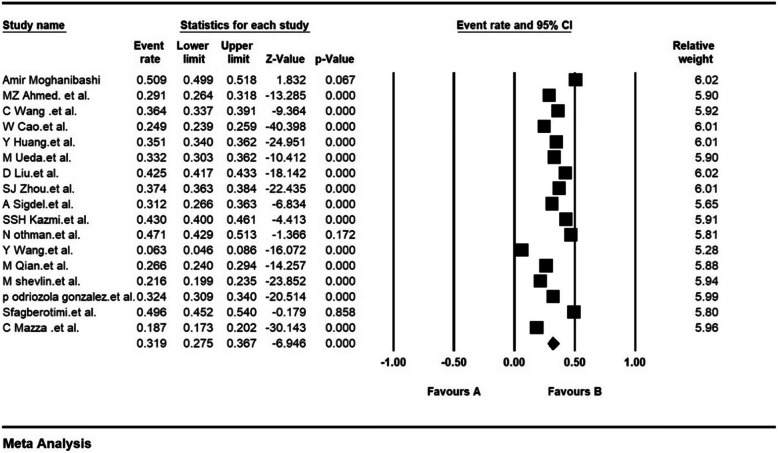
The prevalence of anxiety in the studies based on the random effects model

**Fig. 8:**
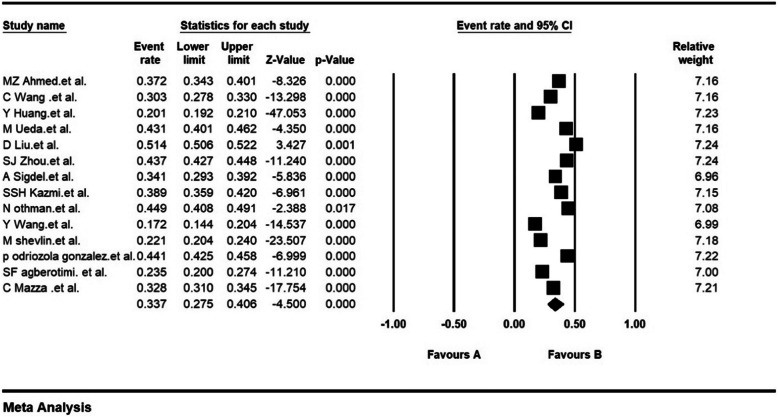
The prevalence of depression in the studies based on the random effects model

The prevalence of stress in 5 of the studies with a sample size of 9074 was 29.6% (95% CI: 24.3–35.4). Results of the 5 studies are evaluated by the Depression, Anxiety and Stress Scale (DASS-21) instrument (Fig. 6). The prevalence of anxiety in 17 studies with a sample size of 63,439 was obtained as 31.9% (95% CI: 27.5–36.7) (Fig. 7). Moreover, the prevalence of depression in 14 studies with a sample size of 44,531 was 33.7% (95% CI: 27.5–40.6) (Fig. 8).

Figures 3, 4 and 5 present the Forest plots for the prevalence of stress, anxiety, and depression based on the random effects model, in which each black square is the prevalence rate, and the length of the line on which the square is located denotes 95% confidence interval. The black diamond shape represents the overall prevalence rate for the symptoms.

### Subgroup analysis

Table 2, reports the prevalence of stress, anxiety, depression among the general population during the COVID-19 pandemic in different continents. The highest prevalence of anxiety in Asia is 32.9 (95% CI: 28.2–37.9), the highest prevalence of stress in Europe is 31.9 (95% CI: 23.1–42.2), and the highest prevalence of depression in Asia is 35.3 (95% CI: 27.3–44.1) (Table 2).

**Table 2:** Investigation of the Prevalence of stress, anxiety, depression among the general population during the COVID-19 pandemic by different continents

Psychological disorders	continents	Number of articles	Sample Size	I^**2**^ (%)	Egger’s test	Prevalence (95% CI)
anxiety	Asia	13	54,596	99.2	0.136	32.9 (95% CI:28.2–37.9)
	Europe	3	8341	98.8	0.272	23.8 (95% CI:16.2–33.5)
depression	Asia	10	35,688	99.5	0.224	35.3 (95% CI:27.3–44.1)
	Europe	3	8341	99.2	0.104	32.4 (95% CI:21.6–45.5)
stress	Asia	3	2758	96.3	0.229	27.9 (95% CI:19.7–37.8)
	Europe	2	6316	98.5	–	31.9 (95% CI:23.1–42.2)

## Discussion

This work is the first systematic review and meta-analysis on the prevalence of stress, anxiety and depression in the general population following the COVID-19 pandemic. This study has followed the appropriate methods of secondary data analysis for examining 17 related research works. The articles used in this study were all cross-sectional. According to our analysis, the prevalences of stress, anxiety, and depression, as a result of the pandemic in the general population, are 29.6, 31.9 and 33.7% respectively.

The emergence of COVID-19, with its rapid spread, has exacerbated anxiety in populations globally, leading to mental health disorders in individuals. This has even caused cases of stereotyping and discrimination [37, 38]. Therefore, it is necessary to examine and recognize people’s mental states in this challenging, destructive and unprecedented time. Evidence suggests that individuals may experience symptoms of psychosis, anxiety, trauma, suicidal thoughts, and panic attacks [39, 40]. Recent studies have similarly shown that COVID-19 affects mental health outcomes such as anxiety, depression, and post-traumatic stress symptoms [22, 24, 31]. COVID-19 is novel and unexplored, and its rapid transmission, its high mortality rate, and concerns about the future can be the causes of anxiety [41]. Anxiety, when above normal, weakens body’s immune system and consequently increases the risk of contracting the virus [39].

Research shows that people who follow COVID-19 news the most, experience more anxiety [39]. Most of the news published on COVID-19 are distressing, and sometimes news are associated with rumors, which is why anxiety levels rise when a person is constantly exposed to COVID-19 news [21]. Misinformation and fabricated reports about COVID-19 can exacerbate depressive symptoms in the general population [23]. The latest and most accurate information, such as the number of people who have improved and the progress of medications and vaccines, can reduce anxiety levels [42]. In this regard, mental health professionals recommend promoting healthy behaviors, avoiding exposure to negative news, and using alternative communication methods such as social networks and digital communication platforms to prevent social isolation [41].

Such conditions are even more significant for populations with poorer health conditions. In the under-developed and developing countriesthe epidemic conditions of COVID-19 impose greater psychological effects on the population, given that these countries are also affected by many other infectious diseases. Uncertainty about health status, follow-up of patients, treatment care, and inefficiency in these communities can also increase the vulnerability of such communities to the psychological effects of COVID-19 [21–36].

The results of epidemiological studies show that women are at a higher risk of depression [43]. Women are more vulnerable to stress and post-traumatic stress disorder than men [44]. In recent studies, the prevalence of anxiety and depression and stress during COVID-19 pandemic is shown to be higher in women than in men [21, 23, 27, 31].

Aging increases the risk of COVID-19 infection and mortality, however, the results of existing studies show that during the pandemic, the levels of anxiety, depression and stress are significantly higher in the age group of 21–40 years. The main reason for this seems to be that this age group are concerned over the future consequences and economic challenges caused by the pandemic, as they are key active working forces in a society and are, therefore, mostly affected by redundancies and business closures [21, 22, 25]. Some researchers have argued that a greater anxiety among young people may be due to their greater access to information through social media, which can also cause stress [45].

During the COVID-19 pandemic, people with higher levels of education had greater levels of anxiety, depression, and stress. According to recent studies, during the COVID-19 pandemic, there is an association between education levels, and anxiety and depression levels [21, 31]. According to a study which was conducted in China, the higher prevalence of mental symptoms among people with higher levels of education is probably due to this group’s high self-awareness in relation to their own health [46]. In addition, anxiety levels are significantly higher in people with at least one family member, relative, or a friend with the COVID-19 disease [21, 24, 42].

Recent studies have revealed an association between medical history and increased anxiety and depression caused by the COVID-19 spread [36]. Previous research works had shown that medical history and chronic illnesses are associated with increased psychiatric distress levels [42, 47]. People who have a history of medical problems and are also suffering from poor health may feel more vulnerable to a new disease [48].

Governments and health officials must provide accurate information on the state of the pandemic, refute rumors in a timely manner, and reduce the impact of misinformation on the general public’s emotional state. These high level activities result in a sense of public security and potential psychological benefits. Governments and health authorities need to ensure that infrastructure is provided to produce and supply adequate amounts of personal protective equipment (PPE), e.g. masks, hand sanitizers and other personal hygiene products during the COVID-19 pandemic. Optimistic and positive thoughts and attitude toward the COVID-19 spread are also protective factors against depression and anxiety [23]. The use of electronic devices and applications to provide counseling can reduce the psychological damages caused by COVID-19, and can consequently promote social stability [31]. The rise in the number of infections and mortalities are likely to affect the symptoms of depression and anxiety. During the H1N1 epidemic, anxiety reached the highest point at the peak of the epidemic and decreased with its decline [49].

Our research has a few limitations; All of the studies in our analysis were periodic, which could reflect the psychological state of the population over a period of time. However, psychological states change with the passage of time and with the alterations in one’s surrounding environment. Therefore, it is necessary to portray the psychological impacts of the COVID-19 catastrophe over a longer and more forward-looking period. Follow-up studies can be helpful in clarifying the mental state of the population in future. Although several research works in this meta-analysis have used the same tests for population screening, yet there were a few studies that followed different scales to assess stress, anxiety and depression.

## Conclusion

In less than a few months, the COVID-19 pandemic has created an emergency state globally. This contagious virus has not only raised concerns over general public health, but has also caused a number of psychological and mental disorders. According to our analysis, it can be concluded that the COVID-19 pandemic can affect mental health in individuals and different communities. Therefore, in the current crisis, it is vital to identify individuals prone to psychological disorders from different groups and at different layers of populations, so that with appropriate psychological strategies, techniques and interventions, the general population mental health is preserved and improved.

## Data Availability

Datasets are available through the corresponding author upon reasonable request.

## Abbreviations

SARS: Severe Acute Respiratory Syndrome
MERS: Middle East Respiratory Syndrome
STROBE: Strengthening the Reporting of Observational studies in Epidemiology
PRISMA: Preferred Reporting Items for Systematic Reviews and Meta-Analysis
